# Intraperitoneal Bladder Rupture Revealed by the Sentinel Clot Sign

**DOI:** 10.5334/jbsr.1517

**Published:** 2018-03-21

**Authors:** Maxime Gudelj, Frederic Giroul, Laurent Dorthu

**Affiliations:** 1 CHR Verviers, BE

**Keywords:** Bladder injury, Clot sign, Abdominal trauma, CT

We present the case of a 44-year old woman with diffuse abdominal pain and abdominal wall tension after a high velocity motor vehicle crash. She was referred for a whole body computed tomography (CT) which showed a substantial amount of free intraperitoneal fluid without injury to liver, spleen, kidneys or bowel. However a spontaneous hyperattenuating structure, the so-called sentinel clot sign was depicted abutting the vesical dome (arrows), more conspicuously on sagittal (Figure 1) and coronal reformations than on axial images (Figure 2). Intraperitoneal bladder rupture was suggested. Bladder catheterization was then performed and revealed a gross hematuria. Surgery confirmed the diagnosis.

**Figure 1:**
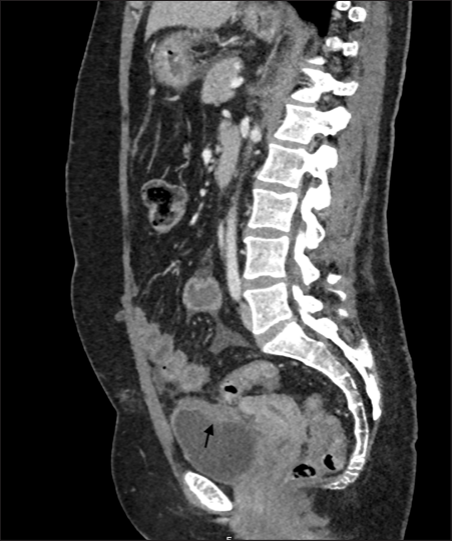
A sagittal reformatted CT image shows a high-attenuating clot abutting on the bladder dome, the so-called « sentinel clot sign ».

**Figure 2:**
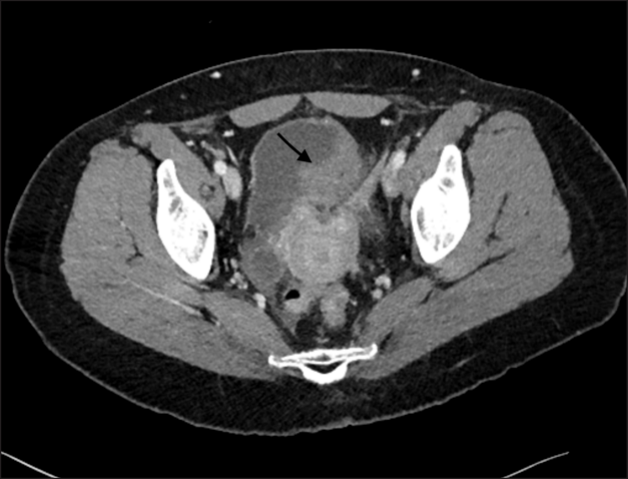
On a transverse CT image, the sentinel clot sign is quite subtle.

## Comment

Bladder injuries occur in about 1.6% of patients with blunt abdominal trauma. Approximately 60% of bladder injuries are extraperitoneal, 30% are intraperitoneal, and the remaining 10% are both extra- and intraperitoneal. Intraperitoneal injuries tend to occur at the dome which is the most vulnerable region of the bladder. Whole body CT is part of the initial evaluation in trauma patients. CT Cystrography can be performed to demonstrate bladder rupture. Nevertheless CT without opacification of the bladder may also depict a sentinel clot sign abutting the bladder dome which indicates injury, with a reported sensibility of 84% [1]. This type of rupture should be promptly diagnosed and treated, owing to the high risks of complications such as peritonitis. This case illustrates the relevance of the “sentinel clot sign” and the importance of orthogonal reformations for improved detection and diagnostic confidence.
